# Efficient ECG Compression and QRS Detection for E-Health Applications

**DOI:** 10.1038/s41598-017-00540-x

**Published:** 2017-03-28

**Authors:** Mohamed Elgendi, Amr Mohamed, Rabab Ward

**Affiliations:** 1 Department of Electrical and Computer Engineering, University of British Columbia, Vancouver, British Columbia Canada; 2 Department of Obstetrics and Gynaecology, University of British Columbia, Vancouver, British Columbia Canada; 3 Department of Computer Science & Engineering, University of Qatar, Doha, Qatar

## Abstract

Current medical screening and diagnostic procedures have shifted toward recording longer electrocardiogram (ECG) signals, which have traditionally been processed on personal computers (PCs) with high-speed multi-core processors and efficient memory processing. Battery-driven devices are now more commonly used for the same purpose and thus exploring highly efficient, low-power alternatives for local ECG signal collection and processing is essential for efficient and convenient clinical use. Several ECG compression methods have been reported in the current literature with limited discussion on the performance of the compressed and the reconstructed ECG signals in terms of the QRS complex detection accuracy. This paper proposes and evaluates different compression methods based not only on the compression ratio (CR) and percentage root-mean-square difference (PRD), but also based on the accuracy of QRS detection. In this paper, we have developed a *lossy* method (Methods III) and compared them to the most current *lossless* and *lossy* ECG compression methods (Method I and Method II, respectively). The proposed *lossy* compression method (Method III) achieves CR of 4.5×, PRD of 0.53, as well as an overall sensitivity of 99.78% and positive predictivity of 99.92% are achieved (when coupled with an existing QRS detection algorithm) on the MIT-BIH Arrhythmia database and an overall sensitivity of 99.90% and positive predictivity of 99.84% on the QT database.

## Introduction

Cardiovascular diseases (CVDs) are cited as the number one cause of death worldwide by the World Health Organization (WHO)^1^. Medical researchers have placed significant importance on cardiac health research, leading to a strong focus on technological advances for cardiac function assessment. One such research pathway is the improvement of the conventional cardiovascular-diagnosis technologies used in hospitals/clinics.

Electrocardiogram (ECG) analysis is the most common clinical cardiac test and has proven to be a useful screening tool for a variety of cardiac abnormalities due to its simple, risk-free, and inexpensive application^2^. The ECG signal contains features that reflect the underlying operation of the heart, and these features represent electrophysiological events that coincide with the sequence of depolarization and repolarization of the atria and ventricles. The signals for each heartbeat contain three main events: the P wave, the QRS complex, and the T wave. Analyzing these events over a short period of time (<30 mins) has been achieved with high accuracy. However, early detection of CVDs requires long-term monitoring using ECG electrodes connected to mobile phones and/or point-of-care devices that rely on wireless communication to improve the development of technological diagnostic devices.

**Figure 1:**
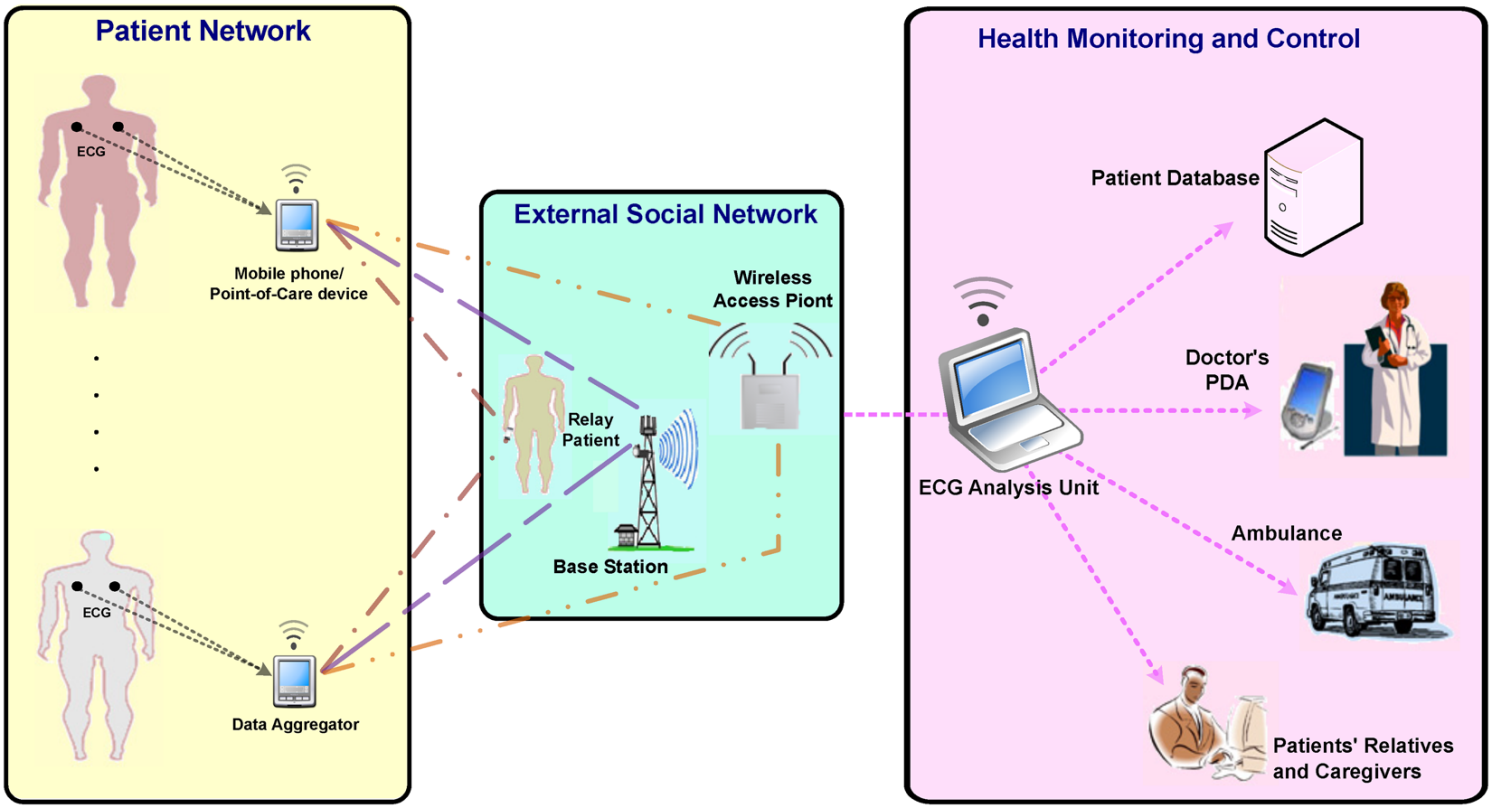
A wireless ECG Monitoring System.

Developing an ECG system that is reliable, scalable, and an effective patient monitoring and medical data management tool is essential for implementing a highly accurate and efficient e-health device for CVD screening and diagnosis. The ECG compression technique proposed in this study leverages current sensors and smartphone technologies for connecting patient networks with a medical infrastructure to facilitate remote patient treatment, as shown in Fig. 1. ECG data aggregators (ECGag) (e.g., mobile phones and point-of-care devices) can be used to acquire, minimally process, and wirelessly transmit ECG signals to an ECG analysis unit (ECGau) (e.g., a device with high computational resources such as a computer). ECGags are usually battery-driven and have limited storage and minimal computational capacity. Thus, a solution that reduces the size of the acquired ECG signals (which are saved, stored, and transmitted by the ECGag) while protecting the integrity of the signal quality (so no information is lost) is needed.

A proper compression method can reduce the size of the transmitted ECG signals. However, most of the high-performance ECG compression methods are not suitable for wireless biosensors because of their complexity^3,4^. Suitable compression methods usually mandate either a low-compression rate or high energy consumption^5^. Therefore, developing a simple, fast, and efficient compression method that is suitable for long-term (≥30 mins) ECG signals is needed. Moreover, most of the ECG compression methods are discussed separately from the ability to detect main events (e.g., QRS complexes).

Based on a review of current literature, the evaluation of the compression method performance is widely evaluated by PRD^6^. Since a highly distorted ECG signal can be useless from a clinical point of view, reporting the impact of compression on detecting QRS complexes is vital. In this paper, we evaluate the overall efficiency of ECG signal transmission in terms of CR, PRD, and QRS detection performance.

Another aspect that will be explored is the local analysis of the ECG signal on the ECGag unit. Developing an algorithm that enables the ECG analysis (such as QRS detection and beat-to-beat interval estimation) to be performed locally on the ECGag is also needed^7,8^. Because the wireless communication between the ECGag and the ECGau unit (cf. Fig. 1) is a major source of power consumption for the ECGag, developing an algorithm that allows for local analysis will reduce the power consumed by the device. Combined with the proposed ECG compression method discussed above, local analysis of the ECG signals will facilitate faster decision making and efficient signal processing with the use of limited resources.

## Results

The performance of the proposed algorithm was evaluated using the MIT-BIH arrhythmia and QT databases. For a fair and consistent evaluation of the proposed method (Method III) with all results published in the literature, the performance of the QRS detection algorithms from each method was assessed using two statistical measures: sensitivity (SE) and positive predictivity (+P). The SE and +P were calculated as follows:

1 $${\rm{SE}}( \% )=\mathrm{TP}/({\rm{TP}}+{\rm{FN}}),$$

2 $$+{\rm{P}}( \% )=\mathrm{TP}/({\rm{TP}}+{\rm{FP}}),$$

In other words, SE reports the percentage of detected true beats out of all true beats, while the +P reports the percentage of detected true beats out of all detected beats. Note, TP stands for true positives (the number of QRS complexes detected as QRS complexes), FN stands for false negatives (the number of QRS complexes which have not been detected), and FP stands for the number of false positives (non-QRS complexes detected as QRS complexes).

Method I demonstrated the performance of the QRS detector with different parameter values. The optimal *W* and *q* values were determined by^8^ after testing various combinations of (*W*, *q*) with *W* ranging from 3 to 6 and *q* ranging from 9 to 17. It was found that optimal detection QRS accuracy was achieved with *W* = 3 and *q* = 15. Note that the optimization step of the QRS detector for Method II was not discussed in ref.^9^.

For Method III, a rigorous optimization over all parameters was conducted. This step is carried out once on either an ECGau or a PC. As soon as the optimal parameters are determined, the ECGag will be adjusted accordingly without any further tuning. As this step occurs one time, it does not add any complexity to the ECG signal analysis at ECGag or ECGau level. The value of *B* varied from *b*_*min*_ = 360 Hz to *b*_*min*_ = 500 Hz, while the value of *K* varied from *k*_*min*_ = 50 Hz to *k*_*min*_ = 360 Hz. As we have multiple objectives, plotting the Pareto frontier (the objective space of possible Pareto solutions) cannot be achieved. Therefore, all Pareto solutions were sorted in descending order according to the overall accuracy (objective function *g*), as shown in Table 1. The optimal Pareto value of *B*/*K* was found to be 4.875, where *B* = 390 Hz and *K* = 80 Hz, achieving a maximum value of *g* = 99.851% with an SE of 99.78% and a +P of 99.92%. It is clear that the highest accuracy of QRS detection was scored using Method III with *B*/*K* = 4.875. The results of Method III will be discussed in more detail.

**Table 1:** Optimal values for Method III based on the QRS detection accuracy.

*B*	*K*	#Beat	TP	FP	FN	SE (%)	+P (%)	*g* (%)
390	80	109985	109775	82	247	99.78	99.92	99.85
390	70	109985	109781	89	249	99.78	99.91	99.85
390	60	109985	109774	88	254	99.78	99.91	99.84
390	110	109985	109748	83	264	99.77	99.92	99.84
380	80	109985	109789	109	238	99.79	99.89	99.84
390	50	109985	109796	110	237	99.79	99.89	99.84
410	80	109985	109705	59	303	99.74	99.94	99.84
380	140	109985	109754	92	257	99.77	99.91	99.84
400	70	109985	109750	81	275	99.76	99.92	99.84
400	60	109985	109750	79	276	99.76	99.92	99.84
.	.	.	.	.	.	.	.	.
.	.	.	.	.	.	.	.	.
.	.	.	.	.	.	.	.	.
.	.	.	.	.	.	.	.	.
.	.	.	.	.	.	.	.	.
.	.	.	.	.	.	.	.	.
500	260	109985	109233	28	803	99.37	99.97	99.67
500	250	109985	109232	28	801	99.37	99.97	99.67
500	300	109985	109223	27	809	99.37	99.97	99.67
500	290	109985	109231	29	808	99.37	99.97	99.67
500	270	109985	109220	31	807	99.37	99.97	99.67
500	310	109985	109209	30	819	99.36	99.97	99.66
500	340	109985	109207	29	822	99.36	99.97	99.66
500	320	109985	109214	29	825	99.35	99.97	99.66
500	330	109985	109201	27	829	99.35	99.97	99.66
500	350	109985	109207	29	827	99.35	99.97	99.66

Results were sorted in descending order according to the overall accuracy (objective function *g*). TP stands for true positives (the number of QRS complexes detected as QRS complexes), FN stands for false negatives (the number of QRS complexes which have not been detected), FP stands for the number of false positives (non-QRS complexes detected as QRS complexes), SE stands for sensitivity, +P stands for positive predictivity, and *g* is the F-score (the harmonic mean of SE and +P).

A summary of the QRS detection results for all recordings using Method III with *B*/*K* = 4.875 is shown in Table 2.

**Table 2:** Performance of Method III using *B*/*K* = 4.875 on the MIT-BIH arrhytmia database.

Record	# of Beats	TP	FP	FN	SE (%)	+P (%)
100	2274	2274	0	0	100.00	100.00
101	1866	1866	2	0	100.00	99.89
102	2187	2187	0	0	100.00	100.00
103	2084	2084	0	0	100.00	100.00
104	2229	2229	5	1	99.96	99.78
105	2602	2601	3	1	99.96	99.88
106	2026	2026	3	0	100.00	99.85
107	2136	2136	0	0	100.00	100.00
108	1763	1786	10	4	99.78	99.44
109	2533	2533	0	0	100.00	100.00
111	2123	2123	0	0	100.00	100.00
112	2539	2539	0	0	100.00	100.00
113	1794	1794	1	0	100.00	99.94
114	1890	1882	2	8	99.58	99.89
115	1953	1953	0	0	100.00	100.00
116	2395	2395	0	0	100.00	100.00
117	1535	1535	0	0	100.00	100.00
118	2278	2278	0	0	100.00	100.00
119	1988	1988	0	0	100.00	100.00
121	1863	1863	0	0	100.00	100.00
122	2476	2476	0	0	100.00	100.00
123	1519	1519	0	0	100.00	100.00
124	1619	1619	0	0	100.00	100.00
200	2601	2601	3	0	100.00	99.88
201	1949	1949	9	0	100.00	99.54
202	2138	2133	0	5	99.77	100.00
203	2988	2965	5	25	99.16	99.83
205	2656	2656	0	1	99.96	100.00
207	2324	2151	3	179	92.32	99.86
208	2953	2946	0	7	99.76	100.00
209	3006	3006	0	0	100.00	100.00
210	2652	2641	1	11	99.59	99.96
212	2748	2748	0	0	100.00	100.00
213	3250	3249	0	1	99.97	100.00
214	2262	2260	0	2	99.91	100.00
215	3362	3362	1	0	100.00	99.97
217	2208	2208	1	0	100.00	99.95
219	2154	2154	0	0	100.00	100.00
220	2048	2048	0	0	100.00	100.00
221	2427	2427	0	0	100.00	100.00
222	2485	2485	2	0	100.00	99.92
223	2604	2604	1	0	100.00	99.96
228	2060	2058	20	2	99.90	99.04
230	2256	2256	0	0	100.00	100.00
231	1571	1571	0	0	100.00	100.00
232	1783	1783	7	0	100.00	99.61
233	3077	3077	1	0	100.00	99.97
234	2751	2751	2	0	100.00	99.93
48 records	109985	109775	82	247	99.78	99.92

The results were obtained using the optimal values of *B* and *K*, which are 390 Hz and 80 Hz, respectively. TP stands for true positives (the number of QRS complexes detected as QRS complexes), FN stands for false negatives (the number of QRS complexes which have not been detected), FP stands for the number of false positives (non-QRS complexes detected as QRS complexes), SE stands for sensitivity, and +P stands for positive predictivity.

**Figure 2:**
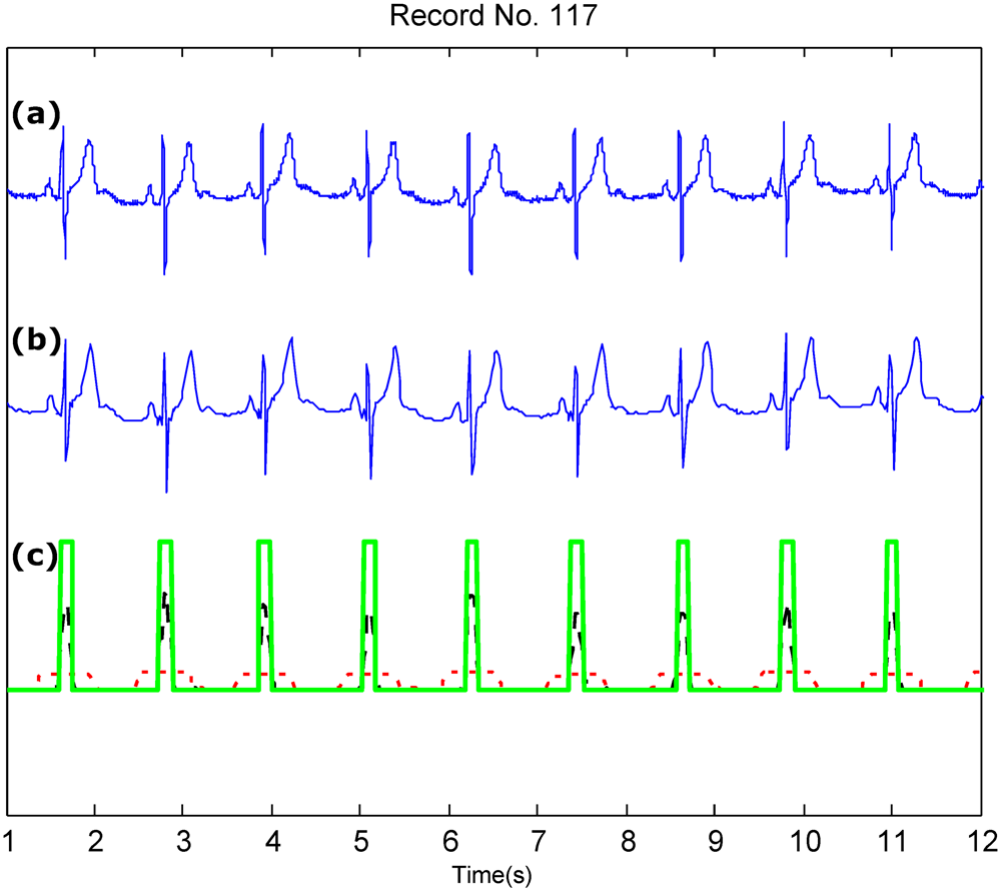
QRS detection over Record 117 of the MIT-BIH Arrhythmia Database with large T waves. (**a**) Original ECG signal (**b**) Compressed ECG signal using Method III (**c**) Compressed ECG signal using Method III with adaptive thresholding and detected QRS complexes (green blocks). Signal amplitudes have been manipulated to fit all signals in one figure. Here, the red dotted line represent the first moving average where the black dashed line represents the second moving average.

**Figure 3:**
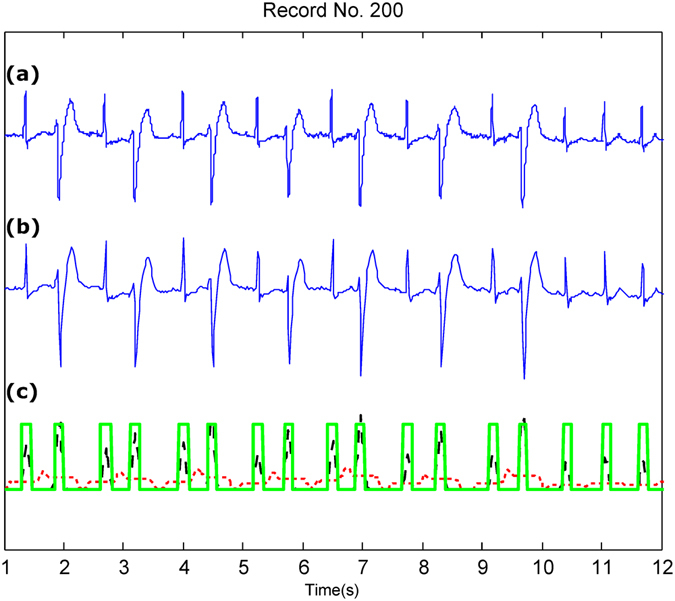
QRS detection over Record 200 of the MIT-BIH Arrhythmia Database with irregular beats (premature ventricular contractions). (**a**) Original ECG signal (**b**) Compressed ECG signal using Method III (**c**) Compressed ECG signal using Method III with adaptive thresholding and detected QRS complexes (green blocks). Signal amplitudes have been manipulated to fit all signals in one figure. Here, the red dotted line represent the first moving average where the black dashed line represents the second moving average.

**Figure 4:**
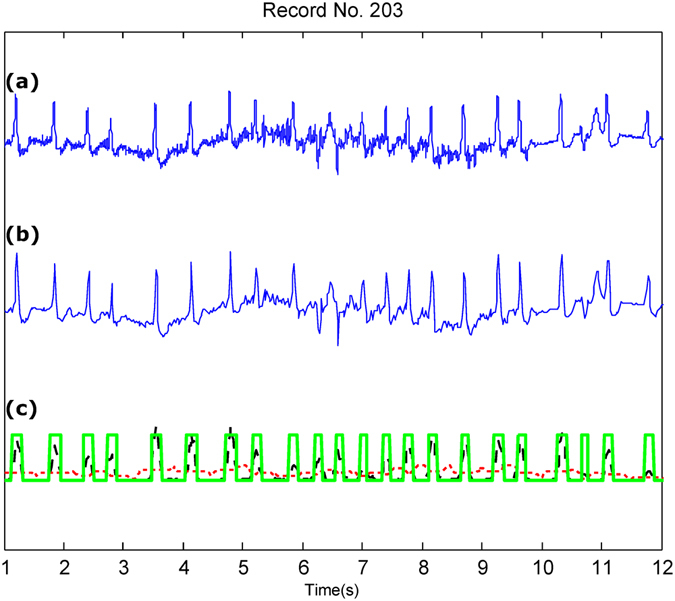
QRS detection over Record 203 of the MIT-BIH Arrhythmia Database with severe baseline drift and noise. (**a**) Original ECG signal (**b**) Compressed ECG signal using Method III (**c**) Compressed ECG signal using Method III with adaptive thresholding and detected QRS complexes (green blocks). Signal amplitudes have been manipulated to fit all signals in one figure. Here, the red dotted line represent the first moving average where the black dashed line represents the second moving average.

Figures 2, 3 and 4 show the performance of Method III under noisy conditions. The first plot (a) in each figure shows the original ECG signal. The second plot (b) shows the compressed ECG signal using Method III. The third plot (c) shows the QRS detection based on the compressed signal shown in plot (b). Figure 2 shows the performance of the QRS detector on a compressed ECG signal using Method III over T waves with large amplitudes, which are often misidentified as QRS peaks due to their amplitude.

Figure 3 shows the QRS detection performance using an ECG signal with premature ventricular contractions, which introduce irregular heartbeats in terms of RR interval and morphology. Figure 4 shows that the QRS detector performed well in the presence of noise and baseline wandering within the compressed ECG signal.

Table 3 compares the QRS detection performance of well-known compression algorithms with the proposed methods. As shown, Method III outperformed all competing *lossless* algorithms in terms of increasing BCR and decreasing PRD simultaneously. The multiscale morphology technique scored lower in detection accuracy given its high computational complexity. In comparison, Method III was fast and more efficient for analyzing ECG signals making it better suited for e-health applications.

**Table 3:** Comparison of the QRS detection with other published algorithms on the MIT-BIH arrhythmia database.

Refs	Method	SE (%)	+P (%)
Chen *et al*.^51^	Wavelet De-noising	99.55	99.49
Poli *et al*.^52^	Genetic Algorithm	99.60	99.51
Afonso *et al*.^53^	Filter Banks	99.59	99.56
Hamilton and Tompkins^54^	BPF/Search-back	99.69	99.77
Zhang and Lian^55^	Multiscale Morphology	99.81	99.80
Ieong *et al*.^56^	Quadratic Spline wavelet	99.31	99.70
Nallathambi and Principe^7^	Pulse Train	99.58	99.55
Martnez *et al*.^57^	Wavelet Delineation	99.66	99.56
Method I	Adaptive Predictor	99.64	99.81
Method II	Compressive Sampling Matching Pursuit	N/R	N/R
Method III	Decimating By A Factor *B*/*K*	99.78	99.92

SE stands for sensitivity, while +P stands for positive predictivity. N/R stands for Not Reported.

Table 4 compares the compression performance of the four methods with other compression schemes implemented on hardware for wearable applications. Method III outperformed existing well-known *lossy* and *lossless* compression methods by scoring the highest BCR at 4.5 while scoring the lowest PRD at 0.53. Note that Methods III and III have already outperformed the state-of-the-art *lossless* compression methods: delta predictor/Rice-Golomb coding^10^, adaptive predictor/Huffman coding^11^, simple predictor/Huffman coding^12^, and slope predictor/fixed-length packaging methods^13^ in terms of increasing BCR and decreasing PRD simultaneously. The higher the BCR and the lower PRD values in an algorithm, the better the compression performance However, the detection of QRS has to be considered when choosing the optimal compression algorithm.

**Table 4:** Compression performance comparison with other algorithms.

Compression Type	Method	Year	# Records Used	BCR	PRD	Refs
Lossless	Simple Predictor/Huffman Coding	2009	N/R	1.92	0	12
	Delta Predictor/Rice Golomb Coding	2011	N/R	2.38	0	10
	Adaptive Predictor/Huffman Coding	2013	N/R	2.43	0	11
	Slope Predictor/Fixed-length Packaging	2013	N/R	2.25	0	13
	Method I	2015	All records in MIT-BIH Arrhythmia DB	2.28	0	8
Lossy	Simultaneous Orthogonal Matching Pursuit	2011	One record from MIT-BIH Arrhythmia DB	7.23	2.57	58
	Compressive Sensing	2011	All records in MIT-BIH Arrhythmia DB	3.44	9	59
	Wavelet Transform	2012	10 records from MIT-BIH Arrhythmia DB	4.0	1.66	60
	Nonuniform Binary Matrices	2012	N/R	5.0	8.58	61
	Compressive Sensing	2012	3 records from MIT-BIH Arrhythmia DB	2.5	2.6	62
	Encoding with Modified Thresholding	2013	4 records from MIT-BIH Arrhythmia DB	5.4	2.7	63
	Compressive Sampling	2013	One record from MIT-BIH Arrhythmia DB	2.5	9	64
	Method II	2015	11 records from MIT-BIH Arrhythmia DB	6.4	3.75	9
	Method III	2017	All records in MIT-BIH Arrhythmia DB	4.5	0.53	—

BCR stands for bit compression ratio, PRD stands for percentage root-mean-square difference, N/R stands for Not Reported, and the symbol ≈ means nearly equal.

**Figure 5:**
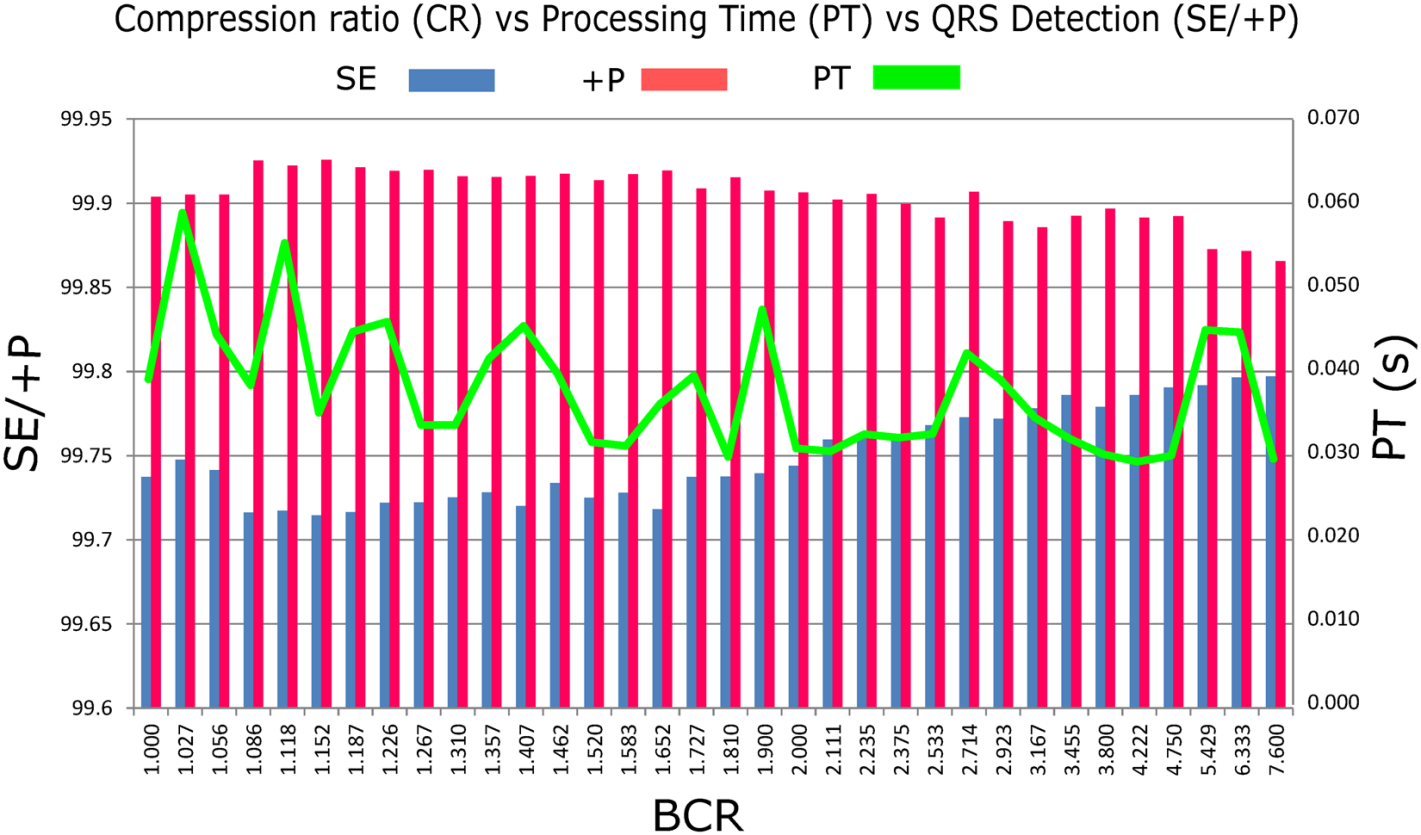
Compression ratio versus performance. Here, BCR stands for bit compression ratio, PT stands for average processing time, SE stands for sensitivity, and +P stands for positive predictivity.

In this study, the proposed detector was implemented in MATLAB 2012a (MathWorks, Inc., Natick, MA, USA) on an Intel™ i5 CPU with 2.27 GHz. Figure 5 shows the BCR versus QRS detection performance and processing time. As expected, the processing time decreased as the BCR increased. Method III took an average processing time of 0.029 seconds with a BCR of 4.5 to compress ECG signals and detect the QRS complexes. The time complexity of Method III is O(N) algorithm, which is similar to the lossy-based compression algorithms^5^, and the complexity is smaller than that of Method I [O(Nlog_2_N)] and Method II [O(Nlog_2_N))]. These results indicate that Method III has quick time response and less resource consumption.

## Discussion

### Methods Implementation

To the best of our knowledge, only one ECG compression method category is extensively discussed in current ECG compression literature; this method is referred to as *lossless*, and can be seen in Method I. The *lossless* ECG compression methodology recovers the original ECG signal exactly as is. However, a second methodology is found in communication signal processing literature that is referred to as *lossy*. With the *lossy* methods, some samples are trimmed off from the original signal. Depending on the type of *lossy* method being used, the eliminated data may or may not be noticeable to the user. Several *lossy* compression methods have been reported in the literature^14–22^. These *lossy* compression methods are strongly embedded in *lossless* compression methods due to the risk of potential distortion^23^.

Based on a scan of current literature, the application of the *lossy* compression method to compress ECG signals, without it being embedded (or part of) in a *lossless*, has *not* yet been investigated. We therefore developed a pure *lossy* method (Method III) and compared it to the most current *lossless* ECG compression method (Method I) and the most current *lossy* ECG compression method (Method II). Note that Method II is not a pure *lossy* compression method, since removal of data bits within a lossless framework is implemented.

In Method I, within the signal regions with steep amplitude variations (such as the QRS complex), the predictor statistics were considerably different and resulted in higher prediction error. Therefore, Deepu *et al*.^8^ used prediction error as a marker to locate the QRS complex in ECG signals. By contrast, Method III does not need the linear predictor step used in Method I.

One advantage of using the prediction error in Method I is that the output has low dynamic range (a smaller amplitude than the original ECG signal) and centers around zero amplitude except for the areas corresponding to the QRS complexes. However, a *lossless* coding scheme is required to preserve the data. The output of the coding step is used to transmit the data instead of transmitting the whole signal to save power/memory resources. It is not required to apply a *lossless* coding scheme for Methods III, because the number of samples that represent the signal have already been reduced; using a *lossless* coding scheme would further reduce the quality of the signal and increase the consumption of power/memory resources unnecessarily.

Coding the output of the four methods can be carried out using variable-length coding schemes like Huffman and arithmetic coding^24^. All four methods are compatible with any of these existing coding schemes. The complexity of encoder/decoder implementation, however, is high for these techniques, although they produced optimal bit representations^24^.

Method I applied a coding-packaging scheme that gives a practical, fixed-length 16-bit output and has low hardware complexity^8^. The coding-packaging routine is based on the two’s complement, which is a mathematical operation on binary numbers, representation of the prediction error signal. As most of the error prediction signal is around zero amplitude, it can be represented with only a few bits. Again, Method III does not need this step, as it will increase complexity and render these algorithms less suitable for wearable devices and e-health applications.

### Compression Performance

It can be seen in Table 4 that *lossless* ECG techniques, such as the delta predictor and Rice-Golomb coding scheme^10^, achieved a maximum BCR of 2.38. However, Rice-Golomb coding is highly complex and requires a dedicated memory^8^. The two-stage adaptive predictor and the Huffman coding schemes^11^ achieved a BCR of 2.43, but Huffman coding is highly complex, generates variable-length coded data, and would need further packaging to interface with a standard input/output^8^. Simple predictor and Huffman coding^12^ achieved a BCR of 1.92. The slope predictor and a fixed-length packaging scheme^12^ achieved a BCR of 2.25. Method I (linear predictor and fixed-length packaging) achieved a BCR of 2.28. On the other hand, the proposed *lossy* compression technique, Method III, achieved a BCR of 4.5.

In general, there are many *lossy* compression techniques to achieve higher BCR but these require complicated signal processing techniques^13^. These approaches also require the usage of more complex hardware, which is not suitable for low-power wearable applications^25,26^, and are therefore not included in the comparison.

Method III achieved a BCR of 4.5 without the need for any packaging. The compression performance of Method III is substantially higher than that in refs^8,10,11^, and Method III validates the compression quality based on the QRS detection accuracy, which is essential for wearable applications. However, ECG compression techniques have typically been applied without being validated based on the QRS detection, making it difficult to assess the quality of the compression technique. Note that although Method III is a *lossy* compression method, the main features of the ECG signal morphology were preserved, as shown in Figs 2, 3 and 4. This indicates that a *lossy* compression method can be sufficient for ECG signal compression, in contrast to *lossless* methods that are more complex and require larger energy consumption.

In a *lossy* such as Method III, the minimum rate at which the ECG signal can be sampled—without losing the main events, such as the QRS complexes—has to be twice the highest frequency to achieve the Nyquist rate [*F*_*s*_ > 2*F*_*max*_]. In other words, the ECG sampling frequency has to be greater than or equal to 40 Hz.

Recent research into the implementation of *lossy* methods in an ambulatory environment faces many challenges^27^. The current *lossy* algorithms, including the compressive sensing algorithms, do not compare favorably with other state-of-the-art lossless compression techniques when considering only CR vs. reconstruction quality^27^. Therefore, the choice of using a *lossy* algorithm depends on its ability to provide a low-power implementation. However, the implementation of *lossy* algorithms are included in *lossless* framework, which adds more complexity to the expected nature of *lossy* algorithms. The main advantage of the proposed Method III, which is a pure *lossy* algorithm, is that it accomplishes a higher compression rate, and higher PRD (higher reconstruction signal quality) while achieving the highest QRS detection rate.

### QRS Detection

The literature cites many QRS algorithms that have not been tested against a standard database, making the results difficult to compare and evaluate. Furthermore, many algorithms scored a high detection performance using the overall number of detected beats (i.e., QRS complexes), as shown in Table 2. Note that the QRS detector in ref.^28^ scored a high overall performance with a SE of 99.89% and a +P of 99.94%. However, the study’s authors excluded files 214 and 215 in the MIT-BIH arrhythmia database^29^, and therefore this algorithm may not be superior in terms of performance. In addition, their algorithm was based on wavelet feature extraction and singularity for classification without applying any compression techniques, which is considered unsuitable for e-health applications.

As noted, some investigators have excluded records from the MIT-BIH arrhythmia database^29^ for the sake of reducing noise in the processed ECG signals; consequently, their algorithms appeared to achieve improved performance. Other researchers excluded segments with ventricular flutter^30^ and signals from patients with paced beats^31^ from their investigations. In contrast, we tested the QRS detector over the compressed ECG signal without excluding any record or particular segment making the results more robust and the algorithm more efficient.

**Figure 6:**
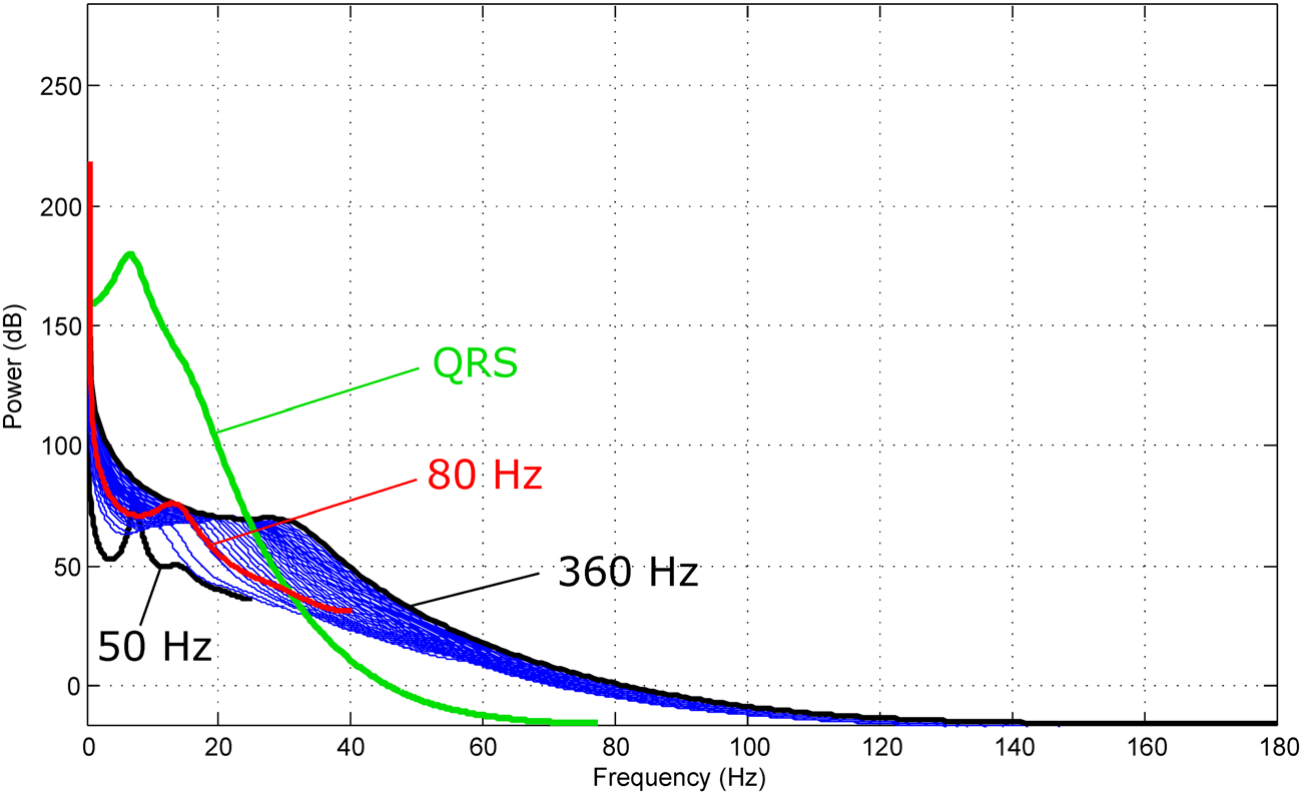
Power spectra of ECG signal (first 60 seconds of record #100 from MIT-BIH Arrhythmia Database). The red curve curve represents the power spectra of Method III with a sampling frequency of 80 Hz. Note, the blue curves represent the power spectra of frequencies between 50 Hz and 360 Hz. The green curve represents the power spectra of QRS complexes (sampled at 360 Hz). It is clear that the optimal frequency band to detect QRS complexes is 0.5–40 Hz.

It is worth noting that Method III achieved a higher QRS detection accuracy because it worked as a filter that captured only the QRS complexes. The evidence of this claim can be seen in Fig. 6, where the main frequencies of the QRS complexes lie in the range of 0.5 Hz to 40 Hz. Method III with a sampling frequency of 80 Hz not only captures the main frequencies of the QRS complexes but also confirms the findings in refs^32–34^.

The detection performance of the Method III on the QT database on a record by record basis is shown in Table 5. The overall comparison of our results with the existing QRS detection algorithms on the QT database is demonstrated in Table 6. It summarizes the performances in terms of number of beats, SE, and +P. Note that the proposed algorithm performed higher in terms of SE and +P when compared to the Pan-Tompkins^33^ and Elgendi^33^ over the same number of beats. It is clear that Method III succeeds in handling long ECG recordings with high performance over the 111,201 automatically annotated heart beats. Moreover, the proposed QRS detector has not been re-tuned, in other words we applied Method III to the QT database without changing the value of any parameter and without re-training the algorithm. The results are promising, and Method III (with the parameters *B* = 390 Hz and *K* = 80 Hz) can be applied over different databases, sampling frequencies, types of arrhythmia, and noise.

**Table 5:** Performance of Method III using *B*/*K* = 4.875 on the QT database.

Record	# of Beats	TP	FP	FN	SE (%)	+P (%)
100	1134	1134	0	0	100.00	100.00
102	1088	1088	0	0	100.00	100.00
103	1048	1048	0	0	100.00	100.00
104	1109	1109	0	0	100.00	100.00
114	867	860	6	10	98.85	99.31
116	1186	1184	0	2	99.83	100.00
117	766	766	0	0	100.00	100.00
123	756	756	0	0	100.00	100.00
213	1641	1639	0	2	99.88	100.00
221	1247	1246	0	1	99.92	100.00
223	1309	1307	0	2	99.85	100.00
230	1077	1077	0	0	100.00	100.00
231	732	732	7	0	100.00	99.05
232	866	866	2	0	100.00	99.77
233	1532	1537	0	0	100.00	100.00
301	1352	1353	0	0	100.00	100.00
302	1501	1499	0	2	99.87	100.00
306	1040	1040	0	0	100.00	100.00
307	853	853	0	0	100.00	100.00
308	1294	1294	0	0	100.00	100.00
310	2012	2007	0	6	99.70	100.00
803	1026	1026	0	0	100.00	100.00
808	903	903	0	0	100.00	100.00
811	704	704	0	0	100.00	100.00
820	1159	1159	0	0	100.00	100.00
821	1557	1555	0	2	99.87	100.00
840	1180	1180	0	0	100.00	100.00
847	803	802	0	1	99.88	100.00
853	1113	1113	0	0	100.00	100.00
871	917	917	0	0	100.00	100.00
872	990	989	0	1	99.90	100.00
873	859	859	0	0	100.00	100.00
883	892	892	0	0	100.00	100.00
891	1267	1267	0	0	100.00	100.00
16265	1031	1031	0	0	100.00	100.00
16272	851	851	0	0	100.00	100.00
16273	1112	1112	0	0	100.00	100.00
16420	1063	1063	0	0	100.00	100.00
16483	1087	1087	0	0	100.00	100.00
16539	922	922	0	0	100.00	100.00
16773	1008	1008	0	0	100.00	100.00
16786	925	925	0	0	100.00	100.00
16795	761	761	0	0	100.00	100.00
17453	1047	1047	0	0	100.00	100.00
104	804	803	0	1	99.88	100.00
106	897	898	0	0	100.00	100.00
107	823	822	0	1	99.88	100.00
110	872	872	4	0	100.00	99.54
111	908	1094	162	10	99.09	87.10
112	684	695	5	0	100.00	99.29
114	698	698	0	0	100.00	100.00
116	560	559	0	1	99.82	100.00
121	1434	1431	0	3	99.79	100.00
122	1414	1414	0	0	100.00	100.00
124	1121	1121	0	0	100.00	100.00
126	945	945	0	0	100.00	100.00
129	672	670	3	8	98.82	99.55
133	840	839	0	1	99.88	100.00
136	810	810	0	0	100.00	100.00
166	813	813	0	0	100.00	100.00
170	897	897	0	0	100.00	100.00
203	1246	1246	0	0	100.00	100.00
210	1063	1063	0	0	100.00	100.00
211	1575	1575	0	0	100.00	100.00
303	1045	1045	0	0	100.00	100.00
405	1216	1216	0	1	99.92	100.00
406	959	959	0	0	100.00	100.00
409	1737	1738	0	0	100.00	100.00
411	1202	1202	0	0	100.00	100.00
509	1028	1028	0	0	100.00	100.00
603	869	869	0	0	100.00	100.00
604	1031	1031	0	0	100.00	100.00
606	1442	1441	0	1	99.93	100.00
607	1184	1183	0	1	99.92	100.00
609	1127	1127	0	0	100.00	100.00
612	751	750	0	1	99.87	100.00
704	1094	1094	0	0	100.00	100.00
30	1018	1016	0	2	99.80	100.00
31	1087	1087	0	0	100.00	100.00
32	1196	1196	0	0	100.00	100.00
33	527	527	0	0	100.00	100.00
34	897	897	0	0	100.00	100.00
35	882	867	3	15	98.30	99.66
36	948	948	0	0	100.00	100.00
37	682	677	2	5	99.27	99.71
38	1563	1563	0	0	100.00	100.00
39	1171	1171	0	0	100.00	100.00
40	1069	1069	0	0	100.00	100.00
41	1366	1366	0	0	100.00	100.00
42	1247	1247	0	0	100.00	100.00
43	1430	1429	0	1	99.93	100.00
44	1337	1333	1	4	99.70	99.93
45	971	971	0	0	100.00	100.00
46	856	856	0	0	100.00	100.00
47	886	886	0	0	100.00	100.00
48	1398	1396	0	2	99.86	100.00
49	833	827	0	6	99.28	100.00
50	661	661	0	0	100.00	100.00
51	749	749	0	0	100.00	100.00
52	1411	1411	0	0	100.00	100.00
17152	1628	1628	0	0	100.00	100.00
14046	1260	1249	0	11	99.13	100.00
14157	1081	1081	0	0	100.00	100.00
14172	663	663	0	0	100.00	100.00
15814	1036	1036	0	0	100.00	100.00
105 records	111201	111323	195	104	99.90	99.84

The results were obtained using the optimal values of *B* and *K*, which are 390 Hz and 80 Hz, respectively. TP stands for true positives (the number of QRS complexes detected as QRS complexes), FN stands for false negatives (the number of QRS complexes which have not been detected), FP stands for the number of false positives (non-QRS complexes detected as QRS complexes), SE stands for sensitivity, and +P stands for positive predictivity.

**Table 6:** Comparison of the QRS detection with other published algorithms on the QT database.

Ref	# Beats	S*E* (%)	+P (%)
Aristotle^57^	86892	97.20	99.46
Martnez *et al*.^57^	86892	99.92	99.88
Pan and Tompkins^33^	111201	97.99	99.05
Elgendi^33^	111201	99.99	99.67
Method III	111201	99.90	99.84

SE stands for sensitivity, while +P stands for positive predictivity.

### Battery-driven ECG Devices

Based on the recommendation in ref.^35^, the better the computational efficiency, the faster the algorithm, and vice versa. Consequently, the faster the algorithm, the more suitable it is for real-time monitoring. In this study we used a computationally efficient QRS detector^33^ along with an optimal compression technique (Method III) to improve both the processing and transmission time.

With advances in computational power, the emphasis on algorithm complexity is slowly decreasing. However, the demand for computationally efficient algorithms still remains for instances where ECG signals are collected and analyzed locally in hospitals, in the home setting, or in remote/rural areas where patient access to hospitals access is limited. Developing a computationally efficient algorithm to accommodate the new trend toward the use of mobile ECG devices is required for these cases. Moreover, implementing a joint compression and QRS detection algorithm to analyze long-term recorded signals in a time-efficient manner is also needed.

Typically, processing long recorded ECG signals is carried out on PCs with efficient memory and high-speed multi-core processors. This advantage is still not available for battery-operated devices: current wearable devices have limited memory and processing power^36–38^. In general, battery-driven ECG devices follow one of three strategies: 1) collect ECG signals for offline analysis; 2) collect ECG signals for real-time analysis within the device itself; or 3) collect ECG signals in real time and analyze the transmitted signals via a remote connection to a separate server. Each strategy has its own advantages and disadvantages in terms of processing time and power consumption. Our proposed Method III can be implemented in each strategy to improve both analysis time and QRS detection accuracy.

### E-health Systems

E-health systems often use ECGags (e.g., mobile phones or personal digital assistants) merely to collect ECG data (either wirelessly or via a wired connection) that are then sent to an ECGau (e.g., a central monitoring station using 4 G mobile telecommunication or internet) for further analysis^39,40^. Applying the proposed compression Method III at the ECGag level is beneficial as it: reduces the transmission delay, saves bandwidth, saves energy on the battery-drived device, saves memory for storage, and speeds up real-time diagnosis feedback. Although some analysis can be done locally on the ECGags before transmitting the compressed ECG signals, the analysis and the subsequent transmission of the ECG signals require a large a mount of energy that is taxes on the ECGag’s limited battery life. Thus, investigating efficient methods for local analysis and transmission of ECG signals is needed in terms of compression and QRS detection. Overall, there is a need for a computationally efficient compression technique and a computationally efficient QRS detector for real-time analysis that must be robust and improve QRS detection accuracy. Simple compression and QRS detection algorithms offer low-cost hardware implementation in both power and size for body sensor networks^41^. Method III can be implemented in the hardware of the ECGag device (or the ECG sensor circuit) and also can be embedded in the software (or an app) of the ECGag device. Because of the robustness, performance, efficiency, and simplicity in implementation, Method III is considered ideal for e-health applications, as it can be implemented on both ECGags and ECGaus.

The proposed method could play a major role in the early detection of disease in low- and middle- income countries (LMICs) where there are major challenges with providing high-quality and universally accessible health care. This is because it follows the framework recommended in ref.^42^ for tackling noncommunicable diseases by achieving simplicity and reliability. Application of the method may increase the capability to develop e-health technologies that significantly impact morbidity and mortality rates, especially for those living in LMICs.

## Conclusions

Our proposed *lossy* compression Method III is a simple yet efficient method that is validated with QRS detection and should be used for wearable, point-of-care, and e-health ECG devices. Method III outperformed existing compression algorithms by achieving a compression ratio of 4.5× with the highest QRS detection accuracy (an SE of 99.78% and a +P of 99.92% using the MIT-BIH arrhythmia database). Results show that Method III is suitable for wearable sensors and processing long-term recordings and large databases as well as for expanding telemedicine capabilities in the near future. To the best of our knowledge, this is the first simple algorithm that improves QRS detection using data compression.

## Methods

### Data Used

The MIT-BIH arrhythmia database, which contains 109,984 heart beats^29^, was used to evaluate the performance of the compression methods. This database is widely used to evaluate ECG compression and QRS detection algorithms as it includes different types of noise and various shapes of arrhythmic QRS complexes^33^. Moreover, the database contains annotation of R peaks for all ECG signals. The benchmark database contains 48 half-hour ambulatory ECG recordings. These recordings have 11-bit resolution over 10 mV and are sampled at 360 Hz. This database is used for training the proposed method and for comparison against the published ECG compression methods.

The QT database with 111,301 beats^43^ is used for evaluating the performance of our proposed compression algorithm. The QT database contains 105 records of 15-minute recording sampled at 250 Hz.

### Compression Method I: Adaptive Linear Prediction

Method I is our benchmark *lossless* compression method to compare and evaluate our proposed *lossy* method against. Several forward–prediction based approaches were used for QRS detection as reported in refs^8,44,45^. Linear forward prediction was used to estimate the current sample *x*[*n*] of the ECG signal in these approaches from its past *m* samples:

3 $$\hat{x}[n]=\sum _{k=1}^{m}{h}^{k}x[n-k]$$

where $\hat{x}[n]$ is the estimate of *x*[*n*], and *h* ^*m*^ is the predictor coefficients. Thus, the prediction error *e*[*n*] (the difference between the actual sample and its estimate $\hat{x}[n]$) is:

4 $$e[n]=x[n]-\hat{x}[n\mathrm{].}$$

**Figure 7:**
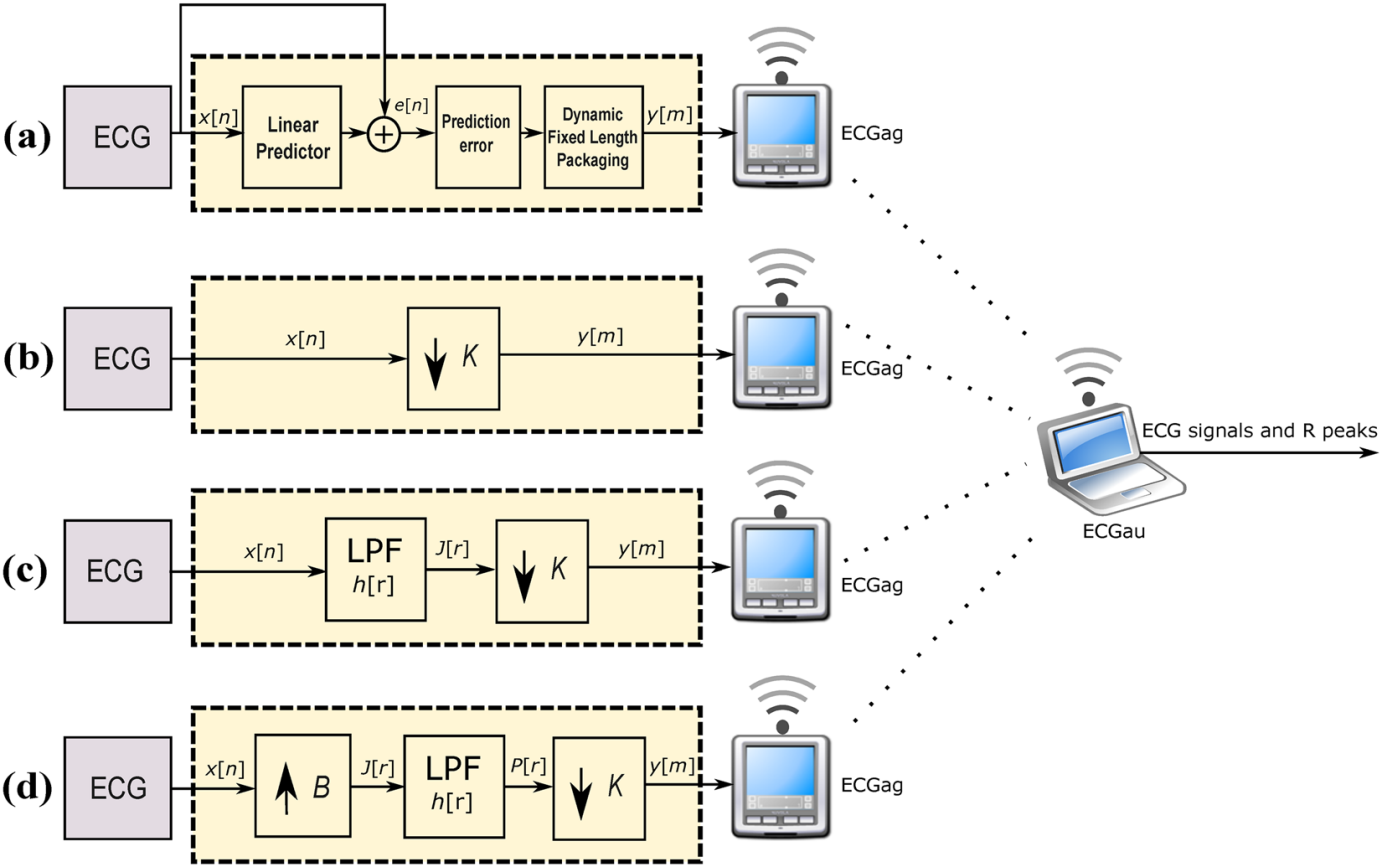
Compression methods. (**a**) Lossless Method I (**b**) Lossy Method II (**c**) Lossy Method III Here, ECGag stands for ECG data aggregators (e.g., mobile phones and point-of-care devices that have limited computational resources that collect the ECG signal) while ECGau stands for ECG analysis unit (e.g. a device with high computational resources such as a computer).

In this paper, we evaluated the recently published work by Deepu and Lian^8^ on ECG compression techniques using adaptive linear prediction. The block diagram representation of this method is shown in Fig. 7(a). The method applied a QRS detector on the prediction error *e*[*n*] signal, followed by fixed-length packaging.

### Compression Method II: Compressive Sampling Matching Pursuit

Method II is our benchmark *lossy* compression method to compare and evaluate our proposed *lossy* method against. It is based on compressive sensing for potential implementation in e-health systems as described in ref.^9^. In Method II the ECG signal goes throw of four processing stages: sampling, redundancy removal module, quantization and Huffman encoding, as shown in Fig. 7(b). The output signal *y*[*n*] is then transmitted to a remote ECGau where the reconstruction of original ECG signal is performed. The novelty of this algorithm relies on the reconstruction algorithm that relies on prior knowledge of ECG wavelet coefficient structure to improve reconstruction quality.

### Compression Method III: Decimating by a Factor *B*/*K*

Method III is our proposed *lossy* compression method. This method achieved a sampling rate conversion by first applying an interpolation step (upsampling followed by a lowpass Filter [LPF]), by factor *B* and then decimating (LPF followed by downsampling) the output by factor *K* as discussed in refs^46,47^. The two filters can be combined as a single LPF with a frequency response *H*(*ω*), which possess the following frequency response characteristic:

5 $$H(\omega )=\{\begin{array}{ll}B, & 0\le |\omega |\le \,{\rm{\min }}(\pi /K,\pi /B)\\ \mathrm{0,} & {\rm{otherwise}}\end{array}$$

where *H*(*ω*) is acting as a LPF for the interpolator and smoothing filter for the decimator. The block diagram for the decimation by a factor *B*/*K* method is shown in Fig. 7(c). The interpolation step can be expressed as follows:

6 $$J[r]=\{\begin{array}{ll}x[r/B], & r=\mathrm{0,}\pm {B},\pm 2{B},\ldots \\ \mathrm{0,} & {\rm{otherwise}}\end{array}$$

Then, the interpolated signal goes to the LPF as follows:

7 $$P[r]=\sum _{l=-\infty }^{\infty }h[r-lB]J[l]$$

After downsampling *J*[*n*] by a factor *K*, the output signal of the decimator is:

8 $$y[m]=P[mK]=\sum _{l=0}^{\infty }h[mK-lB]x[l],$$

where *m* is the data samples of the compressed ECG signals. In other words, if we desire a sampling rate conversion by a ratio *B*/*K* (where *B* and *K* are integers), we can achieve this by first interpolating by *B* and then decimating by *K*. The reason to introduce the interpolation step before the decimation step is to preserve the desired spectral characteristics of the processed signal^48^.

We have two variables *B* and *K* to resample the ECG signal from *B* to *K*. An optimization step is needed to determine the optimal values of *B* and *K*. Any change in these parameters affects the overall performance of the algorithm proposed in this paper. The two variables are interrelated and cannot be optimized in isolation. Our goal is to find the Pareto optimal point, within all possible Pareto solutions^49^ for our multi-objective problem. Our aggregate objective function denoted by *g* is a combination of the three objective functions: T*P*(*B*, *K*), F*P*(*B*, *K*), and F*N*(*B*, *K*) into a scalar function is defined as follows:

9 $$\begin{array}{cccc} & {\rm{argmax}}\,g(B,K) & = & \frac{2\times {\bf{TP}}(B,K)}{2\times {\bf{TP}}(B,K)+{\bf{FP}}(B,K)+{\bf{FN}}(B,K)}\\  & B,K &  & \\ {\rm{subject}}\,{\rm{to}} & {b}_{min}\le B\le {b}_{max}, &  & \\  & {k}_{min}\le K\le {k}_{max}, &  & \end{array}$$

where *g* is the traditional F-measure or balanced F-score, which is the harmonic mean of sensitivity and positive predictivity. TP(*B*, *K*), FP(*B*, *K*), and FN(*B*, *K*) are the three objective functions to be maximized jointly. The Pareto frontier is formed with solutions (the values of two decision variables) that optimizes all parameters. Once the Pareto solutions are achieved, the optimal solution will be used for the implementation. In other words, we are systematically enumerating all possible combinations of *B* and *K* that maximizes the value of *g*. Note, the Pareto optimal solution assures simultaneous improvement of all objectives.

### QRS Detection

The detection algorithm of the QRS complex published in refs^33,35^, a two event-related moving averages (TERMA) algorithm^50^, was used during the data analysis to capture the QRS complexes. TERMA is a fast (computationally efficient) and suitable algorithm for implementation on battery-operated mobile devices, as recommended in refs^33,35^. Therefore, the use of TERMA in combination with the proposed compression algorithm is expected to improve the overall ECG signal analysis, storage capacity, processing time, and signal transmission. In other words, an immediate feedback to the user can be achieved at the ECGag level, long recorded ECG signals can be saved at the ECGags, the transmission between the ECGags and ECGaus will be optimized, and the decision making at the ECGau level will speed up.

The TERMA-based QRS detection algorithm^50^ consists of four stages (filtering, enhancing, generating potential blocks, and thresholding) and uses five parameters (starting frequency [*F*_1_], end frequency [*F*_2_], first moving average [M*A*_*event*_ with a window size of *W*_1_], second moving average [M*A*_*cycle*_ with a window size of *W*_2_], and rejection threshold [*β*]). First, the ECG signal was passed through a third-order Butterworth filter with a bandpass filter *F*_1_ − *F*_2_. The resulting signal was then squared, and two moving averages (M*A*_*event*_ and M*A*_*cycle*_) were applied with a rejection threshold (*β*) to generate blocks of interest. After applying a rigorous optimization step discussed in ref.^33^, the optimal parameters for the QRS detector were *F*_1_ = 8 Hz, *F*_2_ = 20 Hz, *W*_1_ = 97 ms, *W*_2_ = 611 ms, and *β* = 8. Therefore, the QRS detector was within these optimal parameters.

The TERMA-based QRS detector will only be applied to proposed Methods III. Since the QRS detection performance was not reported for Method II, Method I is the benchmark to compare the proposed methods against as described in ref.^8^. Method I will use its already incorporated QRS detector, which removes the high-frequency impulse noise from the prediction error signal. The output will be run through the Savtizky-Golay filter to smooth the incoming signal by approximating the signal within a specified window of size *W* to a polynomial of order *q* that best matched the given signal in a least-squares sense.

### Compression Ratio

The bit compression ratio (BCR) was calculated as follows:

10 $${\rm{BCR}}=\frac{{\rm{size}}({{\rm{BW}}}_{{\rm{u}}})}{{\rm{size}}({{\rm{BW}}}_{{\rm{c}}})},$$

where BW_c_ and BW_u_ refer to the bit widths of compressed and uncompressed samples, respectively. If we evaluate the performance of a compression algorithm based only on BCR, we can conclude that the higher the BCR, the better the compression algorithm.

### Percentage Root Means Squared Difference

The percentage root means squared difference (PRD) is used to quantify the recovered signal quality by measuring the error between original and reconstructed signal, as follows:

11 $${\rm{PRD}}=||x-\hat{x}{||}_{2}\times 100/||\hat{x}{||}_{2},$$

where *x* is raw ECG signal while $\hat{x}$ is the reconstructed ECG signal. If we evaluate the performance of a compression algorithm based only on PRD, we can conclude that the lower the PRD, the better the compression algorithm.
