# Publisher Correction: Efficient ECG Compression and QRS Detection for E-Health Applications

**DOI:** 10.1038/s41598-017-17101-x

**Published:** 2017-12-01

**Authors:** Mohamed Elgendi, Amr Mohamed, Rabab Ward

**Affiliations:** 1 Department of Electrical and Computer Engineering, University of British Columbia, Vancouver, British Columbia Canada; 2 Department of Obstetrics and Gynaecology, University of British Columbia, Vancouver, British Columbia Canada; 3 Department of Computer Science & Engineering, University of Qatar, Doha, Qatar

*Scientific Reports* **7**:459; doi:10.1038/s41598-017-00540-x; Article published online 28 March 2017

This Article contains an incorrect version of Figure 7. The correct Figure 7 appears below as Figure 1.

**Figure 1:**
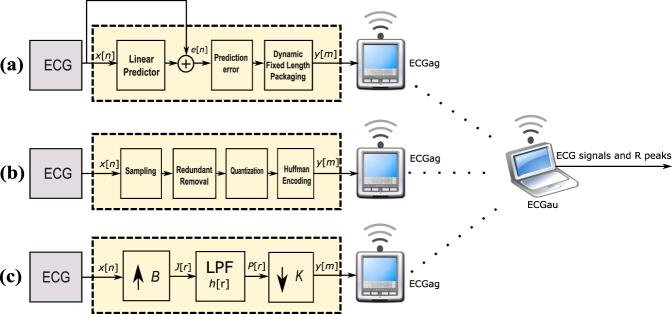
.

